# The Receptor for Advanced Glycation End Products (RAGE) Affects T Cell Differentiation in OVA Induced Asthma

**DOI:** 10.1371/journal.pone.0095678

**Published:** 2014-04-23

**Authors:** Eitan M. Akirav, Octavian Henegariu, Paula Preston-Hurlburt, Ann Marie Schmidt, Raphael Clynes, Kevan C. Herold

**Affiliations:** 1 Research Institute, Islet Biology, Winthrop University Hospital, Mineola, New York, United States of America; and Stony Brook University School of Medicine, Stony Brook, New York, United States of America; 2 Department of Immunobiology, Yale University School of Medicine, New Haven, Connecticut, United States of America; 3 Department of Medicine, New York University School of Medicine, New York, New York, United States of America; 4 Department of Medicine, Columbia University, New York, New York, United States of America; 5 Internal Medicine, Yale University School of Medicine, New Haven, Connecticut, United States of America; Université Libre de Bruxelles, Belgium

## Abstract

The receptor for glycation end products (RAGE) has been previously implicated in shaping the adaptive immune response. RAGE is expressed in T cells after activation and constitutively in T cells from patients with diabetes. The effects of RAGE on adaptive immune responses are not clear: Previous reports show that RAGE blockade affects Th1 responses. To clarify the role of RAGE in adaptive immune responses and the mechanisms of its effects, we examined whether RAGE plays a role in T cell activation in a Th2 response involving ovalbumin (OVA)-induced asthma in mice. WT and RAGE deficient wild-type and OT-II mice, expressing a T cell receptor specific for OVA, were immunized intranasally with OVA. Lung cellular infiltration and T cell responses were analyzed by immunostaining, FACS, and multiplex bead analyses for cytokines. RAGE deficient mice showed reduced cellular infiltration in the bronchial alveolar lavage fluid and impaired T cell activation in the mediastinal lymph nodes when compared with WT mice. In addition, RAGE deficiency resulted in reduced OT-II T cell infiltration of the lung and impaired IFNγ and IL-5 production when compared with WT mice and reduced infiltration when transferred into WT hosts. When cultured under conditions favoring the differentiation of T cells subsets, RAGE deficient T cells showed reduced production of IFNγ but increased production of IL-17. Our data show a stimulatory role for RAGE in T activation in OVA-induced asthma. This role is largely mediated by the effects of RAGE on T cell proliferation and differentiation. These findings suggest that RAGE may play a regulatory role in T cell responses following immune activation.

## Introduction

T cell activation is determined by intrinsic cellular factors consisting of T cell receptor signaling (signal 1) and costimulatory signals (signal 2). However, other extrinsic factors such as cytokines and products of cell death also affect T cell activation, differentiation, and survival by modulating cell intrinsic signals. T cell differentiation may be affected by activating pathways involving recruitment of lck and other signaling molecules. In addition, activation of innate immune receptors and signaling pathways can also modulate T cell differentiation.

Previous studies showed that the receptor for glycation end products (RAGE) plays a role in activation and differentiation of T cells[1], [2], [3], [4]. Blockade of RAGE activation with soluble RAGE attenuated the adoptive transfer of diabetes in NOD mice and also reduced recurrent diabetes in syngeneic islet grafts and islet allograft rejection [1]. This reduction in diabetes was correlated with reduced cellular infiltration of the islet and increased expression of anti-inflammatory cytokines such as IL-10 and TGF-β [1]. Similarly, RAGE blockade results in reduced T cell infiltration of the central nervous system in the experimental autoimmune encephalomyelitis (EAE) model of multiple sclerosis [5]. In transgenic mice expressing a T cell receptor specific for ovalbumin (OVA) (OT-II), the transfer of RAGE-deficient OT-II T cells into RAGE-sufficient hosts resulted in reduced proliferative responses following OVA immunization. This effect was attributable to the expression of RAGE on the OT-II T cells [2].

RAGE also appears to be involved in human immune responses. Patients with chronic obstructive pulmonary disease show increased RAGE expression in the lung and elevated soluble RAGE levels in the bronchial alveolar fluid [5], [6]. Similarly, a recent report demonstrated increased RAGE receptor and ligand levels in asthmatic patients [7], indicating an active role for RAGE in lung inflammation. We reported constitutive expression of RAGE in T cells isolated from diabetic patients but not healthy donors [4].

These previous studies suggest that RAGE can modulate T cell differentiation. Supernatants from cultures with anti-CD3 and anti-CD28 mAbs showed higher levels of IL-10, IL-5, and TNF-alpha secreted from activated RAGE−/− compared with RAGE^+/+^ T cells, while RAGE inhibition in RAGE^+/+^ T cells resulted in reduced production of IFN-γ, suggesting that RAGE may be important in the differentiation of Th1 cell subsets [2], [3]. Furthermore, there was increased expression of RAGE mRNA in clonal T cells activated under Th1differentiating conditions. RAGE−/− T cells showed increased production of Th2 type cytokines including IL5 and IL10 in MLR responses, while RAGE blockade resulted in decreased IFN-gamma production [3]. In vivo stimulation of RAGE deficient OT-II OVA-specific T cells with OVA showed decreased T cell activation and reduced Th1cytokines. This effect was unique to T cells and was independent of RAGE expression on DCs [2].

The reduced Th1 response and increased Th2 response in RAGE deficient mice indicate that RAGE can control T helper differentiation. To further evaluate the effect of RAGE on immune mediated disease, we examined RAGE deficient mice for their ability to respond to a Th2 like response induced with intranasal OVA immunization model of asthma [8]. Our data show reduction in T cell activation and infiltration of the lungs in RAGE deficient mice, and decreased production of both Th1 and Th2 cytokines.

## Materials and Methods

### Mice

Homozygous RAGE-null mice[9] were backcrossed >10 generations into C57BL/6 and were mated with TCR-transgenic mice expressing a TCR that recognizes OVA residues 323–339 in the context of I-A^b^ C57BL/6-Tg(TcraTcrb)425Cbn (OT-II; originally provided by Alan Frey, New York University School of Medicine, New York, NY) [2]. C57BL/6 (termed B6.H2^b^) mice were purchased from The Jackson Laboratory. All animals were maintained in a temperature-controlled room with alternating 12-h light/dark cycles. All experiments were approved by the Institutional Animal Care and Use Committee of Yale University. These studies conform to the Guide for the Care and Use of Laboratory Animals published by the National Institutes of Health (Bethesda, MD).

### Intranasal immunization protocol

WT or RAGE−/− mice were immunized with 100 µg of OVA (Sigma Aldrich) on day 1,2, and 3. Animals received an additional boost of 50 µg OVA intranasally on days 14 and 15 and on days 19 and 20. Mice were sacrificed on day 21 and their tissues were collected for additional analysis. Intranasal immunization of OT-II and OT-II/RAGE−/− was done using 50 µg of OVA on day 1,2, and 3 followed by intranasal boost of 25 µg OVA on days 7, 8, and 9. Mice were sacrificed on day 10 and their tissues were collected for additional studies.

In some experiments, WT or RAGE−/− OT-II cells (107) were transferred into non-transgenic WT C57BL/6 recipients. The mice were then immunized with intranasal OVA as described above and the mediastinal LNs and lungs were isolated as described below. Cell counts were performed and the cells were analyzed for expression of Vβ5.1/5.2 by flow cytometry as described below.

### Bronchial alveolar and lung collection

Bronchoalveolar lavage (BAL) was performed by cannulation of the trachea and lavage with 1 ml of PBS. Total cell counts were performed, and cells isolated for further analysis. Lungs were collected and were process for either histology or FACS analysis as described below.

### T cell cultures

To assess the effects of RAGE on skewing of T cells, we stimulated purified Tcells from WT or RAGE−/− B6 mice with plate bound anti-CD3 antibody (10 ug/ml) plus soluble anti-CD28 antibody (1 ug/ml) for four days under the following skewing conditions: for Th1 anti-IL4 antibody (5 ug/ml), IL12 (3.5 ng/ml) and IL2 (20 U/ml), for Th2 anti IFNγ antibody (5 ug/ml), IL2 (20 U/ml), IL4 (10 ng/ml) and for Th17 both anti IL4 and anti IFNγ antibodies plus IL23 (20 ng/ml), IL6 (20 ng/ml), and TGF beta (0.5 ng/ml). After four days of stimulation, the cells were harvested from the wells, washed and replated in uncoated wells for an overnight rest. The following day the resting cells were harvested, run through a ficoll separation before counting and replating in wells coated with anti-CD3. The cells were re-stimulated for 24 hr and the supernatants were collected and frozen at −80 until analysis by Luminex assay or by Elisa. For these assays, antibodies and cytokines were obtained from Dr. Richard Flavell's laboratory.

### Immunofluorescence staining

Lungs from OVA immunized mice were perfused using PBS, mounted in OCT compound (Tissue-Tek) and snap frozen in liquid nitrogen. Frozen tissues were sectioned at 7 µm thickness and fixed using acetone. Hematoxylin staining was done to evaluate lung infiltrating mononuclear cells. PE-conjugated rat anti-mouse CD4 antibody (BD Pharmingen) was used to detect T cell infiltration in OT-II transgenic mice.

### FACS analysis

BAL fluid, lungs, and mediastinal LNs were analyzed by FACS (FACSCalibur BD Biosciences). The following mAbs were used for T cell analysis: rat anti-mouse CD4, rat anti-mouse CD8a, anti-CD3e, allophycocyanin-conjugated rat anti-mouse CD25, anti-CD62L, and anti-TCR Vβ5.1/5.2 (all from BD Pharmingen). Granulocytes in the BAL were detected using rat anti-mouse GR-1 (BD Pharmingen).

### Cytokine measurement

Supernatants were removed from in vitro cultures of lymph node cells harvested from primed mice and frozen until analyzed. The cells were stimulated with or without OVA peptide and Luminex assays were performed on the supernatants using the Th1/Th2 cytokine 6plex panel (Invitrogen) or a murine 8plex kit from Millipore. Supernatants were analyzed for the following analytes: IL2, IL4, IL5, IL10, IL12, IL13, IL17, and IFNγ, following the kit protocols and assays were read on a Biorad Bioplex analyzer (Biorad). In studies of T cell skewing, IFNγ was analyzed by ELISA.

### Statistical Analyses

Unless otherwise indicated mean±SEM are shown. All comparisons between WT and RAGE−/− cells were done with Student's t-test using GraphPad Prism 5.

## Results

### Intranasal OVA treated RAGE deficient mice show reduced cellular infiltration in the lung

**Figure 1:**
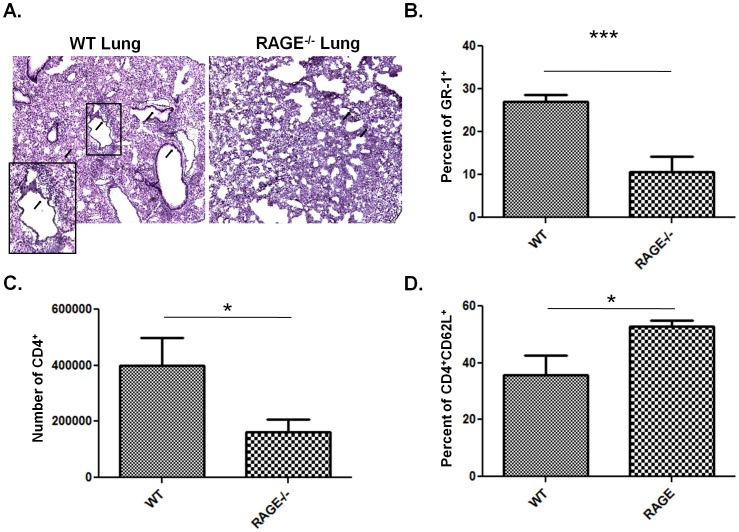
Cellular infiltration of the lung is reduced in OVA immunized RAGE−/− mice. WT or RAGE−/− mice were challenged with intranasal OVA as described in Materials and Methods. *(* ***A*** *)* H&E staining of lungs of WT and RAGE−/− mice. *(* ***B*** *)* Percent of Gr-1^+^ cells in the bronchial alveolar lavage fluid of OVA immunized mice (n = 5 per group, ***p<0.003). *(* ***C*** *)* Absolute numbers of CD4^+^ in the lungs of OVA immunized mice (WT: n = 15; RAGE−/−: n = 14; *p<0.045). *(* ***D*** *)* Percent of CD4^+^CD62L^+^ cells in the mediastinal LNs of OVA immunized mice (WT: n = 4, RAGE−/−: n = 5; *p<0.035).

Previous reports in both human and mouse show an increase in RAGE expression during acute lung injury and a stimulatory role for RAGE in murine T cell activation[10]. We therefore examined whether RAGE deficiency affects overall immune activation and cellular infiltration in mice immunized with intranasal-OVA. Hematoxylin and eosin staining of lung sections from WT or RAGE−/− revealed an accumulation of mononuclear cell in the lungs and airways of WT mice that was reduced in the lungs of RAGE−/− mice (Figure 1A). FACS analysis of the cellular infiltrates from WT and RAGE−/− mice showed a significant reduction in the numbers of GR-1^+^ cells (Figure 1B, p<0.003), and CD4^+^ T cells (Figure 1C, p<0.045) in the BAL fluid and lungs of RAGE−/− mice, respectively. In the mediastinal lymph nodes, there were increased CD62L expressing CD4^+^ cells in RAGE−/− mice when compared with WT controls (Figure 1D, p<0.035). These data suggest that RAGE expression is required for normal immune activation and cellular infiltration in the lungs of OVA treated mice.

### OVA specific RAGE−/− OT-II cells show reduced IFNγ and IL-5 production following OVA challenge

**Figure 2:**
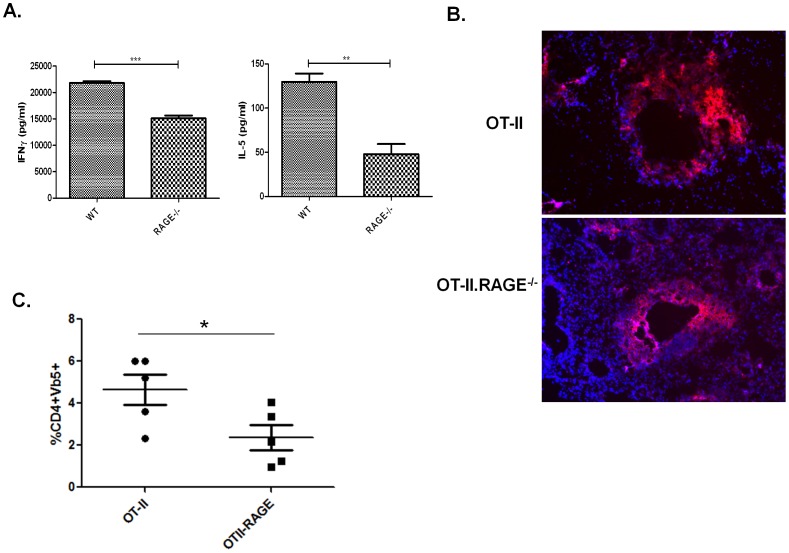
OVA specific OT-II CD4^+^Tcells showed reduced cytokine production and lung infiltration in OVA immunized mice. RAGE sufficient or deficient OT-II TCR-Tg mice were immunized with intranasal OVA as described in Materials and Methods. *(* ***A*** *)* Luminex analysis of IFN-γ (left panel) and IL-5 (right panel) levels in the supernatant of OVA stimulated OT-II cells derived from mediastinal LNs of WT or RAGE−/− mice immunized with OVA. The levels of cytokines in unstimulated cultures were less than the lower limit of detection. Results from a single experiment representative of experiments with 3 and 4 WT and RAGE−/− OT-II mice respectively are shown (*p<0.05). IF staining of CD4^+^ (red) and DAPI (blue) cells *(* ***B*** *)* and percent of infiltrating CD4^+^Vβ5^+^ cell *(* ***C*** *)* (OT-II: n = 5, OT-II.RAGE−/−; n = 5; *p<0.04) in the lungs of OVA immunized OT-II and OT-II.RAGE−/− mice. We did not find T cell infiltration in animals that had not been previously exposed to OVA.

To more specifically examine the role of RAGE in mediating T cell activation, we tested the effects of RAGE deficiency on lung infiltrates of OVA-specific OT-II TCR transgenic T cells, and their responses to OVA *in vitro* following OVA stimulation and priming *in vivo*. RAGE deficient or sufficient OT-II mice were immunized intranasally with OVA. Following activation, mediastinal LNs were collected and cells were tested in a recall response assay. Luminex analysis of supernatant of OVA stimulated RAGE−/− OT-II cells showed reduced production of IL-5, and IFNγ (Figure 2A) and IL-10 (not shown). Immunofluorescence staining of the lungs showed an accumulation of CD4^+^ cells in the airways of both OT-II and RAGE−/−OT-II mice (Figure 2B). There was a significant reduction in the percentage of CD4^+^Vβ5^+^ OT-II cells in the lungs of RAGE−/− mice when compared with control mice (Figure 2C, p<0.042). Overall these data demonstrate that RAGE deficient OVA specific CD4 T cells show reduced cytokine production by cells from draining lymph nodes and reduced cellular accumulation in the lung.

**Figure 3:**
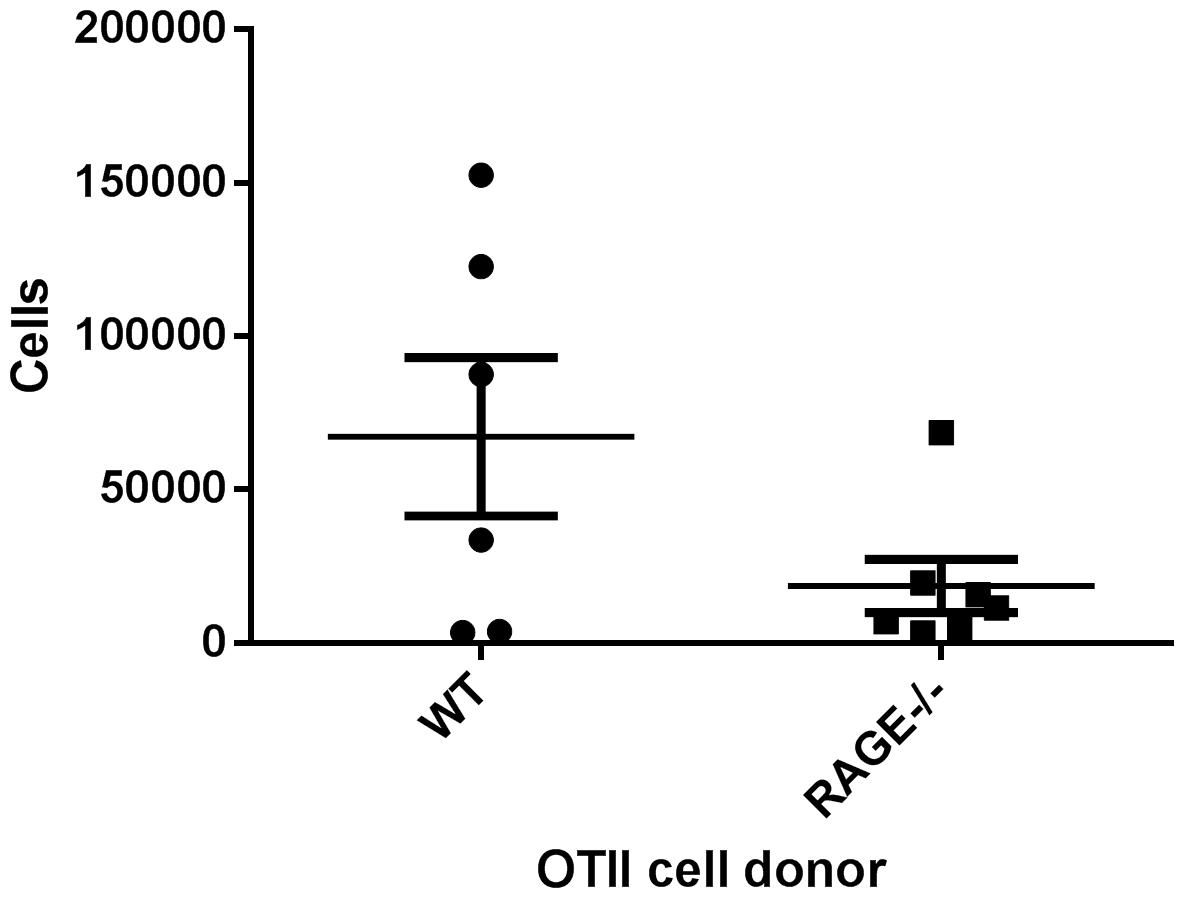
RAGE−/− OT-II T cells show reduced infiltration in lung when transferred into WT hosts. Adoptive transfer experiments were performed as described in Materials and Methods with OT-II T cells from WT (n = 7) or RAGE−/− (n =  6) mice. The total number of cells were counted in the lungs after gating on CD4+Vβ5.1/5.2+ T cells that were detected by flow cytometry. There were reduced numbers of Vβ5.1/5.2^+^ T cells in the recipients of RAGE−/− OT-II T cells (p = 0.08).

RAGE is highly expressed on pulmonary tissue [11]. In order to determine whether the behavior of the RAGE−/− T cells was due to effects on T cells or were the result of RAGE deficiency on the pulmonary tissue, we transferred WT or RAGE−/− OT-II transgenic T cells into WT non-transgenic hosts and challenged the mice with intranasal OVA. There was a 43% reduction of the OT-II cells in the draining lymph nodes and a reduced numbers of OT-II T cells in the BAL fluid of the mice that had received RAGE−/− T cells (Figure 3, p = 0.08).

### RAGE affects activation and differentiation of T cells

**Figure 4:**
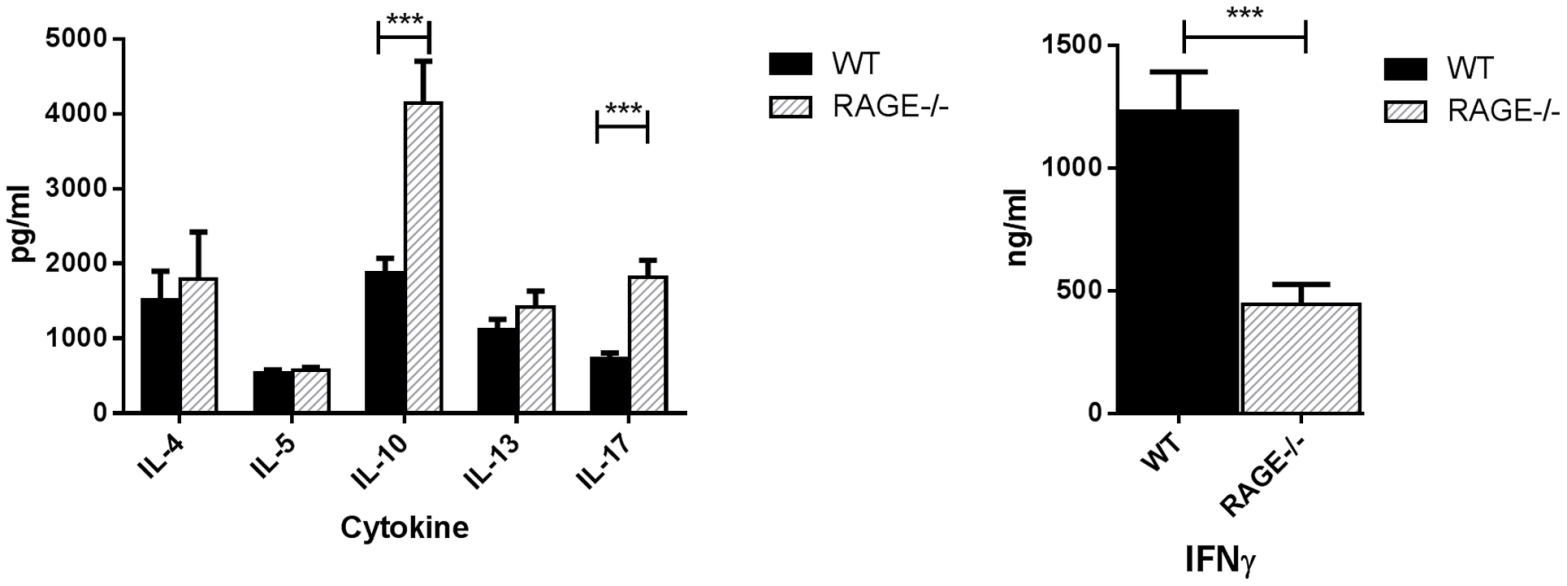
RAGE deficiency reduces Th1 while enhancing Th17 differentiation in T cells. Purified T cells from WT or RAGE−/− mice were cultured under conditions that favor differentiation into Th1, Th2, or Th17 cells as described in Materials and Methods. After 4 days of primary stimulation and one day of resting, cells were restimulated overnight with plate bound anti-CD3. The levels of cytokines in the supernatants were measured by Luminex or by ELISA. The skewing of the cell phenotypes was verified by measurement of secreted cytokines in the cultures of WT cells. The levels of IL-17 produced when the T cells were cultured withIL23, IL6 and TGF-β were increased in the RAGE−/− T cells vs WT whereas the levels of IFNγ were reduced when the cells were cultured with anti-IL-4 mAb and IL12 (p<0.0001). (n = 9 to 10 each, WT = black bar, RAGE−/− = gray bar).

Our studies of OT-II cells showed reduced cytokine production, Therefore, we sought to examine whether RAGE expression could alter the differentiation into T cells into different phenotypes. To address this, we cultured T cells from WT or RAGE−/− mice in conditions that directed differentiation into Th1, Th2, or Th17 T cells. We confirmed the ability of the different culture conditions to skew WT T cell differentiation, followed by a comparison of cytokine production in WT and RAGE−/− T cells cultured under the cytokine-skewing conditions. When cultured in the presence of anti-IFNγ mAb and IL-4, we found similar production of IL-4, IL-5, and IL-13 by T cells from RAGE−/− and WT mice, but increased production of IL-10 (Figure 4). However, there was significantly lower levels of production of IFNγ from T cells cultured in the presence of IL-12 and anti-IL-4 mAb (p<0.001) but greater production of IL-17 when the cells were cultured with IL23, IL-6 and TGFβ (p<0.001). The levels of IL-2 were similar under all culture conditions in WT and RAGE−/− cells (25.6±2.19 pg/ml and 25.9±2.05 pg/ml in WT and RAGE−/− cells respectively under Th1 skewing conditions.) These studies suggest that there is a selective role of RAGE on differentiation of T cells.

## Discussion

The receptor for advanced glycation end products has been implicated in T cell activation and differentiation through direct and indirect evidence. Removal of RAGE ligands with soluble RAGE can attenuate adaptive immune responses and RAGE−/− mice and their T cells show reduced functional responses. However, it has not been clear whether all cells are similarly affected by RAGE, the previous studies had suggested a predominant effect on Th1 responses. We therefore tested the role of RAGE in OVA induced lung inflammation, a model of human asthma, and evaluated the effects of RAGE deficiency on T cell activation. Our data show that RAGE is required for T cell activation and cytokine production in this model following intranasal OVA immunization and there were reduced responses that included IFNγ, IL-5, and IL-10 production. RAGE deficient mice showed reduced T cell infiltration of the lung and decreased granulocyte and antigen specific T cell accumulation in BAL fluid. When we studied RAGE−/− T cells under conditions that cause skewing of T cells subsets we found reduced levels of IFNγ but increased levels of IL-17 from RAGE−/− T cells.

We have previously shown that T cell activation was greatly impaired in MLR reactions of T cells from RAGE deficient mice or following TTP488 (a small molecule inhibitor of RAGE) [3]. This reduction was associated with impaired proliferation and diminished IFN-γ and TNF-α production. In contrast, Th2-type cytokines, such as IL5 and IL4, were increased in the absence of RAGE, suggesting that RAGE may enhance Th2 immunity in mice [3]. In addition, when T1D was adoptively transferred into NOD/scid recipients by diabetogenic splenocytes, there was increased expression of IL-10 and TGF-β in the pancreases when the recipients were treated with soluble RAGE.

However, in this setting, we found decreased expression of both Th1 and Th2 cytokines by the pathogenic OVA-II T cells but our studies of T cells differentiation indicate that there is not a specific effect of RAGE on differentiation of Th2 cells. Instead, our studies now performed under culture conditions that lead to T cell skewing show an effect on Th1 cell differentiation. This is similar to our previous studies showing a direct role for RAGE expression on T cells that when cultured with RAGE−/− or WT dendritic cells Similarly differences in cytokine levels in supernatants from mixed lymphocyte reactions or following activation with anti-CD3 and anti-CD28 mAb have now been shown to depend on the RAGE status of the Tcells [2], [3]. The effects of RAGE deficiency on priming of Th17 cells had not previously been studied. The differences between our findings on cytokine production in vivo and in vitro under conditions of T cell skewing may reflect the more complex role of RAGE in T cell activation that manifests as reduced total cytokine production in the setting of antigen exposure in vivo. Indeed, in previous studies, we found reduced proliferative responses of RAGE−/− T cells in mixed lymphocyte reactions as well as reduced proliferative responses of RAGE−/− T cells in vitro[2], [3].

RAGE is also expressed on pulmonary tissues and recent studies have suggested that RAGE and its ligands may play a role in obstructive pulmonary disease [11]. A potential limitation, of our studies, therefore, is that the reduced expression of RAGE on tissue cells could have modified the T cellular responses, perhaps even explaining the changes in cytokine secretion that we observed. However, our adoptive transfer studies of WT or RAGE−/− OT-II T cells suggest that there is a direct effect of RAGE on T cell responses that does not require the participation of tissue RAGE. Furthermore, our functional studies of T cells suggest that there were differences in the responses of WT and RAGE−/− T cells.

These new observations add to studies in other experimental settings and with human cells showing a consistent effect of RAGE on the differentiation of T cells. The basis for this effect is not known since studies to address the role of RAGE on early activation events on T cells have not been done. Collectively these and previous studies indicate that the effects of RAGE on T cell differentiation involves events in T cell differentiation proximal to STAT signaling. We compared Erk signaling in RAGE−/− and WT T cells and did not identify a difference in this signaling molecule, but evidence from other experimental systems have shown a role of RAGE-heparin sulfate and RAGE-HMGB1 complexes on cell signaling [12], [13], [14]. Signaling through RAGE involves interactions between the FH1 domain of mammalian Diaphanous-1 that interacts with the cytoplasmic tail of RAGE and can involve several intermediaries including NF-κB, MAPKs, PI3K/Akt, Rho GTPases, Jak/STAT, and Src family kinases [15]. Thus several activation pathways may be affected by RAGE signaling. Further studies will be required to identify those pathways specifically affected that may lead to activation and differentiation of T cells.

This report further illustrates a more general role of RAGE in T cell activation than had been appreciated from previous studies that had suggested effects that were limited to Th1 cells. These new findings, together with our previous studies that showed RAGE expression on unstimulated T cells from patients with diabetes, indicate that its role may be extensive. Additional studies on a molecular basis will be needed to address these unresolved questions regarding T cell activation.
